# How exosomal platelet-derived miRNAs can lead to spontaneous osteoclastogenesis in osteoporosis: a new mechanistic viewpoint

**DOI:** 10.3389/fmed.2025.1720672

**Published:** 2025-11-26

**Authors:** Francesca Salamanna, Gianluca Giavaresi, Alberto Di Martino, Agostino Gaudio, Fabiana Nucera, Cesare Faldini, Milena Fini

**Affiliations:** 1 Surgical Sciences and Technologies, IRCCS Istituto Ortopedico Rizzoli, Bologna, Italy; 2 1st Orthopaedic and Traumatologic Clinic, IRCCS Istituto Ortopedico Rizzoli, Bologna, Italy; 3 Department of Biomedical and Neuromotor Science-DIBINEM, University of Bologna, Bologna, Italy; 4 Department of Clinical and Experimental Medicine, University of Catania, Catania, Italy; 5 Scientific Direction, IRCCS Istituto Ortopedico Rizzoli, Bologna, Italy

**Keywords:** exosomal platelet-derived miRNAs, spontaneous osteoclastogenesis, osteoporosis, platelet, platelet-bone-axis

## Abstract

Osteoporosis is a chronic bone disease characterized by impaired bone remodeling and increased fracture risk. While classical mechanisms implicate estrogen deficiency, aging, and altered receptor activator of the nuclear factor-κB ligand (RANKL)/osteoprotegerin (OPG) signaling, growing evidence supports a pivotal role of immune and inflammatory pathways in sustaining osteoclast-mediated bone resorption. A distinctive hallmark observed in osteoporotic patients is spontaneous osteoclastogenesis (SO), defined as the ability of mononuclear precursors to differentiate into osteoclasts even in the absence of exogenous stimuli such as RANKL or macrophage colony-stimulating factor (M-CSF), a process driven by an intrinsically primed *in vivo* microenvironment that includes platelets. We hypothesize that platelets may contribute to this priming not only through soluble mediators but also via the release of extracellular vesicles, particularly exosomes enriched in regulatory microRNAs (miRs). Within this framework, platelet-derived exosomal miRs (P-EXO-miRs) may orchestrate multiple intercellular interactions within the bone marrow microenvironment, modulating monocytes, macrophages, stromal and endothelial cells, as well as T and B lymphocytes. Specifically, miR-21, miR-223, miR-214, and miR-155 emerge as key candidates capable of regulating cytokine secretion, inflammatory signaling, and the RANKL/OPG balance, thereby promoting a pro-osteoclastogenic milieu. Network-based analysis using miRNet further supports the involvement of these miRs in pathways such as Hedgehog, Wnt, and actin cytoskeleton regulation, all relevant to osteoclast differentiation and function. Through these mechanisms, P-EXO-miRs may amplify chronic low-grade inflammation and facilitate spontaneous osteoclast differentiation and activity, ultimately contributing to bone loss in osteoporosis. Future investigations should aim to experimentally validate this platelet–bone axis, delineate the molecular targets of individual miRs, and explore their potential as circulating biomarkers or therapeutic targets. By unveiling this previously unrecognized role of platelet-derived miRs in SO, this hypothesis opens new perspectives for the understanding, early detection, and treatment of osteoporosis.

## 1 Introduction

Osteoporosis is a chronic, systemic bone disease characterized by low bone mass, deterioration of bone microarchitecture, and an increased risk of fragility fractures (1, 2). It affects hundreds of millions of people worldwide, predominantly postmenopausal women and elderly individuals, and is a major cause of disability and morbidity (3, 4). The pathophysiology of osteoporosis has traditionally been viewed through the lens of imbalanced bone remodeling, whereby bone resorption by osteoclasts exceeds bone formation by osteoblasts, leading to net bone loss (1, 2). Although hormonal factors (e.g., estrogen deficiency), nutritional status, and aging are key contributors, increasing evidence highlights the pivotal role of immune and inflammatory mechanisms in exacerbating osteoclast-mediated bone resorption (5–7).

An emerging phenomenon during osteoporosis is spontaneous osteoclastogenesis (SO), defined as the differentiation of peripheral blood mononuclear cells (PBMCs) into mature, multinucleated, bone-resorbing cells in the absence of exogenous factors, e.g., receptor activator of nuclear factor kappa-B ligand (RANKL) or macrophage colony-stimulating factor (M-CSF) (8–10). This “spontaneous” behavior reflects the persistence of *in vivo* priming signals that predispose circulating monocytes toward osteoclast commitment. SO has been observed in cultures of PBMCs from osteoporotic patients and is thought to result from an “intrinsically primed” bone microenvironment (11). This environment is enriched in pro-inflammatory cytokines such as tumor necrosis factor-α (TNFα), interleukin-1β (IL-1β), and IL-6, and is further influenced by altered RANKL/osteoprotegerin (OPG) expression and by the activity of immune cells such as activated T lymphocytes and macrophages (11). Together, these factors lower the activation threshold for osteoclast precursors, enabling osteoclastogenesis even in the absence of canonical stimuli.

Nonetheless, other factors may contribute to the modulation of, or be associated with, SO in this pathological setting. Growing evidence suggests a positive correlation between platelet levels and decreased bone mineral density (BMD) associated with osteoporosis (12, 13). Platelets, long recognized for their role in hemostasis and thrombosis, have gained attention also as versatile regulators of immunity and tissue remodeling (14–16). Beyond releasing growth factors and cytokines upon activation, including platelet-derived growth factor (PDGF), transforming growth factor-β (TGF-β), CCL5 (RANTES), and CXCL12 (SDF-1), platelets can also influence local and systemic inflammatory processes (13). Platelet activation is often increased in aging, metabolic disorders, and chronic inflammatory states, all of which overlap with the risk factors for osteoporosis (16).

In recent years, platelets have also emerged as a source of extracellular vesicles (EVs), particularly exosomes (30–150 nm), which carry bioactive molecules such as proteins, lipids, and nucleic acids, especially microRNAs (miRs) (17). Platelets release exosomes during activation or apoptosis, contributing to intercellular communication and modulation of biological processes (17). Although anucleate, platelets retain the cytoplasmic machinery necessary for miR maturation, enabling them to process precursor miRs into mature forms. Key enzymes such as Dicer and Ago2 are functionally active, and complexes like Ago2–miR-223 have been shown to regulate important platelet targets, including P2Y12 (18). These findings indicate that platelets are not passive carriers of megakaryocyte-derived miRs but have the intrinsic ability to generate mature miRs. Recent in-depth transcriptional analyses have identified up to 532 different miRs and as many as 3000–6000 mRNA in human platelets (19, 20), among which the most abundantly expressed include miR-21, miR-155, miR-214, and miR-223 (21). A substantial body of research has progressively demonstrated the profound biological roles of platelet-derived miRs, which not only regulate platelet protein synthesis but can also be transferred to recipient cells, thereby modulating gene expression and impacting both physiological and pathological processes (19–21).

Recently we hypothesized that platelet-derived pro-inflammatory cytokines may indirectly stimulate SO, thereby contributing to the loss of bone mass associated with osteoporosis (11). Additionally, we propose that the interaction between platelets and endothelial cells, macrophages, and other immune cells, all key players in bone resorption and pathophysiology of osteoporosis, could mediate this relationship. Here, we build upon previous discussions and propose that P-EXO-miRs, released by activated platelets in osteoporotic conditions, may provide an additional stimulus for SO. Exploring this innovative vision of platelet–bone axis, which was not addressed prior, may provide new insights into SO in osteoporosis and open avenues for innovative diagnostic and therapeutic strategies.

## 2 Platelet-derived exosomal-miRs

Platelets are the second most abundant blood cell type and possess three principal types of secretory organelles, α-granules, dense granules, and lysosomes, which collectively store a wide range of bioactive molecules. Alpha-granules, the most abundant, contain over 280 proteins, including von Willebrand factor (vWF), platelet factor 4 (PF4), P-selectin, and PDGF (22, 23). Dense granules are enriched in small signaling molecules such as calcium, ADP/ATP, and serotonin (5-hydroxytryptamine) (24), while lysosomes, though less abundant, store acid hydrolases, cathepsins, and lysosomal membrane proteins (25).

Beyond the classical soluble mediators, a substantial pool of extracellular vesicles (EVs) is stored in platelet granules and released upon activation and apoptosis (26). Among these, exosomes, small lipid bilayer vesicles of 30–150 nm, originate from the endosomal system through the inward budding of late endosomal membranes, leading to the formation of multivesicular bodies (MVBs) that subsequently fuse with the plasma membrane to release their contents (23, 27–29). Their biogenesis is regulated by the endosomal sorting complexes required for transport (ESCRT) machinery and associated proteins such as ALIX and TSG101 (30, 31). Once secreted, exosomes serve as key mediators of intercellular communication, transferring bioactive cargos, including microRNAs (miRs), messenger RNAs, proteins, lipids, and metabolites, to recipient cells and thereby influencing gene expression, differentiation, and functional behavior.

The surface of exosomes bears characteristic membrane proteins, including tetraspanins (CD9, CD63, CD81), integrins, adhesion molecules, and proteoglycans, which together determine their stability, cellular tropism, and uptake mechanisms (32). These features confer a high degree of targeting specificity, allowing exosomes to operate as a finely tuned communication network within complex tissue environments. In compartmentalized niches such as the bone marrow, exosomes can traverse the extracellular matrix and vascular barriers to deliver concentrated molecular messages to osteogenic, stromal, endothelial, and immune cells (32). This capacity for localized and systemic signaling renders them particularly relevant in disorders marked by aberrant bone remodeling, such as osteoporosis.

Platelet-derived exosomes (P-EXOs) constitute the predominant exosomal population in circulation, accounting for more than 70% of plasma EVs (27). Their molecular composition reflects the activation state of their parental platelets, which are exquisitely responsive to inflammatory, metabolic, and mechanical cues. P-EXOs carry a broad repertoire of cargos, including mRNAs, proteins, lipids, and, notably, miRs, that can modulate gene expression in recipient cells (23, 28–34). Among these, miRs are small non-coding RNAs (18–25 nucleotides) that regulate up to two-thirds of human genes by promoting mRNA degradation or inhibiting translation (33). Their intrinsic stability against endogenous ribonucleases enables efficient communication across both short and long distances (33, 34). Consequently, P-EXO-miRs represent a versatile system linking platelet activation to systemic regulation of gene expression. Their abundance, cargo diversity, and dynamic modulation under pathological conditions suggest a potential role as both biomarkers and effectors of altered bone remodeling. It is therefore plausible that P-EXO-miRs contribute to the dysregulation of bone homeostasis observed in osteoporosis and SO.

Given the relevance of P-EXO-miRs in intercellular communication, it is important to clarify also what makes them distinct among circulating exosome populations. Unlike exosomes released from other circulating or stromal cell types, P-EXOs constitute the quantitatively predominant vesicle population in human plasma, accounting for more than half of all circulating exosomes (35). This abundance, combined with their high reactivity to physiological stimuli, positions platelets as a major hub of intercellular communication (36). The molecular composition of P-EXOs mirrors that of their parental platelets, which are uniquely sensitive to inflammatory, metabolic, and mechanical cues (37). In contrast to leukocyte- or endothelial-derived exosomes, P-EXOs are particularly enriched in hemostatic proteins, lipid mediators, and specific miRs (miR-21, miR-223, miR-126, miR-146a), which collectively reflect platelet activation states (19–21).

In the context of osteoporosis and other age-related inflammatory conditions, increasing evidence indicates that platelets undergo functional remodeling (12–16). Hyperactivation of platelets has been reported in postmenopausal and senile osteoporosis, accompanied by elevated levels of circulating platelet-derived vesicles and altered miR signatures (12–16, 38). For example, upregulation of pro-inflammatory miRs such as miR-21, miR-155, and miR-223 has been observed in osteoporotic subjects, suggesting that platelet-derived miRs may serve as systemic indicators of the chronic inflammatory and oxidative milieu characteristic of bone loss (39). Moreover, these P-EXOs can interact with immune and stromal cells in the bone marrow, transferring such osteoimmunomodulatory miRs to influence osteoclast differentiation and activity.

Taken together, the unique quantitative predominance, dynamic responsiveness, and disease-associated remodeling of P-EXOs distinguish them from other exosomal sources, making them compelling candidates as both mediators and biomarkers of the SO processes observed in osteoporosis.

## 3 Hypothesized link between P-EXOs-miRs and spontaneous osteoclastogenesis in Osteoporosis

In osteoporosis, platelets are thought to become activated and release P-EXOs (35). These exosomes carry regulatory miRs (P-EXOs-miRs), which may contribute to remodeling the bone marrow microenvironment and promoting SO.

Efficient delivery of P-EXOs to the bone marrow niche likely depends on molecular recognition between exosomal surface proteins and adhesion receptors on target cells (40). Platelet-derived exosomes are enriched in tetraspanins (CD9, CD63, CD81), integrins (αIIbβ3, αVβ3), and P-selectin, all of which may mediate selective binding to activated endothelium, stromal cells, and mononuclear phagocytes. Under osteoporotic conditions, chronic low-grade inflammation increases endothelial permeability and upregulates adhesion molecules such as ICAM-1, VCAM-1, and selectins, potentially enhancing P-EXO recruitment to the marrow (41, 42). Moreover, chemokines such as CCL2 and CXCL12, typically elevated in osteoporosis, may act as molecular “beacons” guiding P-EXOs to specific niches where osteoclast precursors reside (43, 44). Once in proximity, these vesicles are internalized by endocytosis, phagocytosis, or membrane fusion, releasing their miR cargo and initiating downstream signaling cascades that culminate in SO.

**FIGURE 1:**
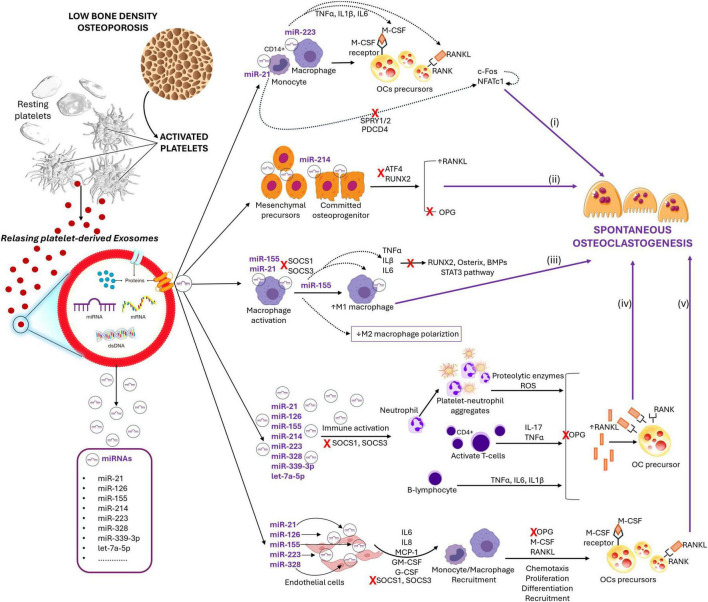
Schematic representation of hypothesized link between P-EXO-miRs and SO in osteoporosis. Arrows (black) indicate activation; blunt-ended lines (red) indicate inhibition. (i) miR-223 as exosomal mediator of hematologic–immune–bone crosstalk; (ii) exosomal miR-214 and the paracrine modulation of the RANKL/OPG equilibrium by stromal and osteoblastic cells; (iii) exosomal miR-155 as a driver of pro-inflammatory macrophage activation and osteoclastogenesis; (iv) platelet-derived miRs and immune-mediated osteoclastogenesis; (v) platelet-derived miRs and endothelial-mediated osteoclastogenesis.

Figure 1 summarizes the principal cellular and molecular interactions hypothesized, which can be organized into five synergistic pathways contributing to SO.

**(i) miR-21 and miR-223 as exosomal mediators of hematologic–immune–bone crosstalk**

P-EXOs are efficiently internalized by CD14^+^ monocytes and bone marrow macrophages, where they deliver miR cargos that modulate transcriptional programs converging on canonical osteoclastogenic pathways, with miR-21 and miR-223 emerging as particularly relevant mediators (45). First, miR-21 is thought to exert a pro-osteoclastogenic role by targeting and suppressing the expression of inhibitory molecules such as programmed cell death 4 (PDCD4) and sprouty homologs 1 and 2 (SPRY1/2) (46, 47). PDCD4 functions as a negative regulator of osteoclast differentiation by repressing the activation of transcription factors that are indispensable for this process, most notably nuclear factor of activated T cells cytoplasmic 1 (NFATc1) (46, 47). In parallel, SPRY1/2 act as endogenous antagonists of the MAPK/ERK signaling cascade, thereby limiting downstream induction of c-Fos and other osteoclastogenic effectors (46–48). The concerted suppression of these inhibitory checkpoints by miR-21 would thus relieve the transcriptional blockade on both the MAPK/ERK–c-Fos axis and the NFATc1 pathway. Consequently, miR-21 could facilitate the synchronized upregulation and functional cooperation of c-Fos and NFATc1, which together constitute the master regulators of osteoclast lineage commitment and differentiation (48). Within this hypothesis, P-EXO miR-21 may therefore act as a molecular amplifier of osteoclastogenesis, shifting the bone marrow microenvironment toward a pro-resorptive state that contributes to bone loss in osteoporosis. In parallel, miR-223 is proposed to contribute to osteoclastogenesis primarily through its capacity to modulate cytokine secretion by macrophages. This miR has been implicated in the regulation of transcripts controlling the synthesis and release of key pro-inflammatory mediators, including TNF-α, IL-1β, and IL-6 (46, 49). These cytokines are well-established regulators of osteoclast differentiation and activity, acting through both direct and indirect mechanisms (49). Specifically, TNF-α can directly enhance osteoclast precursor differentiation and survival, while also indirectly promoting osteoclastogenesis by stimulating RANKL production in stromal cells and osteoblasts (49). IL-1β, in turn, may potentiate osteoclast activity not only by direct stimulation of mature osteoclasts but also by upregulating RANKL synthesis in neighboring cells (49). IL-6 further contributes to the osteoclastogenic milieu by activating multiple pathways, including the induction of RANKL expression and the amplification of inflammatory signaling cascades (49). By regulating the transcription or stability of mRNAs encoding these cytokines, miR-223 delivered by P-EXOs could therefore shape a pro-inflammatory and pro-resorptive bone marrow environment (50–53). Within this framework, miR-223 acts as an immunomodulatory switch that enhances the paracrine crosstalk between macrophages, stromal cells, and osteoclast precursors, thereby reinforcing the conditions necessary for spontaneous osteoclast differentiation in osteoporosis (48, 51, 52). Taking together, these mechanisms support the hypothesis of functional intercellular communication between platelets, macrophages, and bone compartment, whereby exosomal miRs orchestrate a crosstalk among the hematologic system, the immune system, and bone metabolism, ultimately promoting spontaneous osteoclast differentiation and activation.

**(ii) Exosomal miR-214 and the paracrine modulation of the RANKL/OPG equilibrium by stromal and osteoblastic cells**

Beyond their effects on monocytic and macrophagic precursors, P-EXOs may also interact with stromal cells and osteoblasts, two pivotal cellular components of the bone marrow niche that orchestrate osteoclastogenesis by finely tuning the balance between receptor activator of RANKL and its soluble decoy receptor OPG (54). In this context, the transfer of miR-214 via P-EXOs may exert a dual and convergent impact on the osteoblastic compartment (54, 55). On one hand, miR-214 has been shown to suppress the expression of activating transcription factor 4 (ATF4) and runt-related transcription factor 2 (RUNX2), both of which are indispensable for osteoblast differentiation and function. Their repression would compromise the capacity of stromal cells and osteoblasts to mature and sustain bone formation (55). On the other hand, miR-214 may directly downregulate OPG expression, thereby eliminating a crucial inhibitory checkpoint that restrains RANKL-mediated osteoclast activation (54, 55). The combined effects of impaired osteoblastogenesis and reduced OPG production would ultimately shift the RANKL/OPG ratio in favor of RANKL. This imbalance would create a microenvironment intrinsically permissive to osteoclast differentiation and activity, even in the absence of acute inflammatory or hormonal triggers (54–56). Thus, miR-214 carried by P-EXOs emerges as a potential molecular link between defective bone formation and excessive bone resorption, amplifying the pathogenic cascade that underlies SO in osteoporosis.

**(iii) Exosomal miR-155 as a driver of pro-inflammatory macrophage activation and osteoclastogenesis**

An additional critical mechanism for SO may involve the indirect activation of the inflammatory bone marrow microenvironment mediated by P-EXOs. In particular, the transfer of miR-155 to resident macrophages has the potential to skew their polarization toward a pro-inflammatory M1 phenotype, thereby enhancing the secretion of cytokines such as TNF-α, IL-1β, and IL-6 (57). These cytokines are well-recognized cofactors in osteoclastogenesis, as they not only promote the differentiation, survival, and resorptive activity of osteoclasts but also exert profound inhibitory effects on the osteoblastic compartment (50, 58–60). Specifically, TNF-α has been shown to suppress osteoblast differentiation by downregulating essential transcription factors, including RUNX2, Osterix, and members of the BMP family, while simultaneously inducing apoptosis in mature osteoblasts and reducing the synthesis of extracellular matrix proteins such as type I collagen (50). Similarly, IL-1β impairs bone formation by inhibiting matrix mineralization, repressing the expression of osteogenic genes such as ALPL and COL1A1, and stimulating the production of reactive oxygen species in stromal cells, further compromising osteoblast viability and function (50). IL-6, in turn, has been implicated in diverting mesenchymal stem cell commitment away from the osteoblastic lineage toward adipogenesis (50). In addition, IL-6 can directly suppress the activity of mature osteoblasts through activation of the STAT3 pathway, ultimately leading to reduced osteocalcin synthesis (50). Beyond these paracrine effects, miR-155 also acts intracellularly by targeting suppressor of cytokine signaling 1 (SOCS1) and SOCS3, two critical negative regulators of JAK/STAT signaling (61). Under physiological conditions, SOCS1 and SOCS3 function as endogenous brakes that limit the magnitude and duration of cytokine responses (61, 62). Their inhibition by miR-155 would unleash sustained STAT activation, reinforcing the pro-inflammatory state of macrophages and stromal cells (63). This prolonged signaling not only stabilizes M1 macrophage polarization but also potentiates IL-6/STAT3 activity in stromal and osteoblastic cells, further impairing osteoblastogenesis and bone matrix synthesis (61–63). Through this combination of SOCS1/3 suppression and cytokine-mediated paracrine effects, P-EXO–delivered miR-155 may therefore act as a central amplifier of bone marrow inflammation and bone resorption, reinforcing the conditions that underlie SO in osteoporosis.

Nevertheless, given the pleiotropic nature of miR-155 signaling, it is important to acknowledge that its biological role is highly context-dependent. While several studies identify miR-155 as a pro-osteoclastogenic mediator through the promotion of inflammatory macrophage polarization, other evidence suggests that it can also exert regulatory or inhibitory effects on osteoclastogenesis, depending on the cytokine and metabolic context (49). In the setting of osteoporosis, however, chronic low-grade inflammation and oxidative stress are likely to favor the pro-inflammatory and osteoclastogenic functions of miR-155, amplifying the positive feedback loops between immune activation and bone resorption.

**(iv) Platelet-Derived miRs in the crosstalk between immunity and osteoclastogenesis**

Several miRNAs carried by platelet-derived exosomes (including miR-21, miR-155, miR-214, miR-223, and let-7a-5p) may be also internalized by resident immune cells in the bone marrow, such as neutrophils, T lymphocytes, and B lymphocytes, thereby modulating their phenotype and function toward a pro-inflammatory state (64, 65).

Activated platelets interact with neutrophils to form stable aggregates that promote the release of proteolytic enzymes (e.g., elastase, cathepsins) and the production of reactive oxygen species (ROS) (64). In addition to sustaining local inflammation, these platelet–neutrophil aggregates are major sources of neutrophil extracellular traps, which further concentrate proteases and oxidants at the bone–immune interface. This oxidative and proteolytic microenvironment enhances activation of the RANK/RANKL axis, either by directly stimulating osteoclast precursors or indirectly through the recruitment and activation of additional immune cells (66). Moreover, oxidative stress can activate transcription factors such as NF-κB and NFATc1 in pre-osteoclasts, accelerating their differentiation into mature bone-resorbing cells (66, 67). In parallel, proteases and ROS remodel the extracellular matrix, not only degrading structural components but also liberating matrix-bound growth factors (e.g., TGF-β, IGFs), which further support osteoclastogenesis (68).

Helper T-cell subsets, particularly Th17 cells, play a pivotal role in the regulation of bone resorption through their release of pro-inflammatory cytokines (68). Among these, IL-17 and TNF-α are potent mediators of osteoclastogenesis, acting both directly on osteoclast precursors and indirectly by stimulating stromal and osteoblastic cells to enhance RANKL expression (68). Notably, the osteoporotic microenvironment, enriched in IL-6, IL-1β and TNF-α, may further promote Th17 polarization, thereby amplifying local pro-osteoclastogenic signals (69). A key upstream event is Th17 cell polarization, i.e., the differentiation of naive CD4 + T cells into Th17 effectors (69). This process is driven by a specific cytokine milieu: TGF-β provides the baseline signal, while inflammatory cytokines such as IL-6, IL-1β, and IL-21 amplify the differentiation cascade, with IL-23 stabilizing the Th17 phenotype (50, 69). The transcription factor RORγt is the master regulator of this lineage, while additional modulatory signals, such as oxygen tension, microbiota-derived metabolites, and the aryl hydrocarbon receptor (AhR) pathway, further tune the magnitude of Th17 responses (70). Within this context, exosomal miRs, particularly miR-155, have emerged as critical modulators of immune polarization. By targeting transcriptional and signaling pathways in CD4 + T cells, miR-155 favors a shift toward Th1/Th17 phenotypes, thereby amplifying the release of IL-17, TNF-α, and other pro-osteoclastogenic mediators (68). These cytokines not only promote the differentiation and activation of osteoclast precursors but also create a feedback loop that sustains inflammation and bone resorption.

Under inflammatory conditions, B cells become an alternative source of RANKL and cytokines such as TNF-α, IL-6, and IL-1β (71). Beyond their canonical role in antibody production, activated B cells can therefore function as key immunomodulators of bone remodeling, directly promoting osteoclast differentiation. In osteoporosis and other chronic inflammatory states, B cells often shift toward a pro-inflammatory phenotype, driven by cytokine signaling and miR regulation. MiRs including miR-223 and miR-214 have been implicated in modulating B-cell activation and cytokine profiles, favoring the release of osteoclastogenic mediators (72, 73).

The convergence of these events results in increased local RANKL availability, reduced OPG levels, and the establishment of a persistent inflammatory microenvironment that favors spontaneous osteoclast differentiation even in the absence of external stimuli. Moreover, the functional reprogramming of neutrophils, T cells, and B cells not only amplifies pathological bone resorption but also embeds these immune populations into the broader network of immune–skeletal interactions, thereby further consolidating the concept of osteoimmunology.

Nonetheless, as observed for miR-155, both miR-21 and miR-223 exhibit context-dependent biological activities that can shift according to the inflammatory and metabolic milieu. MiR-223 has been shown to act as a negative regulator of osteoclast differentiation under physiological conditions, whereas in inflammatory or hypoxic environments it can promote osteoclast precursor activation (74). Likewise, miR-21 has been reported to influence both osteogenic and osteoclastogenic pathways depending on the signaling background (75). Within the chronic pro-inflammatory and oxidative microenvironment typical of osteoporosis, persistent platelet and immune activation likely bias the function of these miRs toward a net pro-osteoclastogenic outcome. This context-dependent regulation highlights the dynamic nature of exosomal miRNA-mediated communication in bone remodeling.

**(v)** **Platelet-Derived miRs shaping endothelial contributions to osteoclastogenesis**

Bone marrow endothelial cells, particularly those lining the sinusoids, represent highly sensitive targets of P-EXOs, even under physiological conditions. Exosome internalization is facilitated by endothelial receptors such as CD36 and integrins and induces a functional remodeling of endothelial cells toward a pro-inflammatory and chemoattractive state, resulting in the production of cytokines, chemokines, and hematopoietic growth factors (76, 77). In this context, several P-EXO–derived miRs may contribute in a complementary manner. miR-155, through direct repression of SOCS1, promotes macrophage polarization toward an M1 phenotype, prolongs activation of the JAK/STAT1 pathway, and enhances the secretion of pro-inflammatory cytokines such as TNF-α and IL-6 (57, 78). Similarly, miR-21 suppresses SOCS1 and SOCS3, thereby amplifying inflammatory responses in both endothelial cells and macrophages, whereas miR-126 regulates vascular permeability and increases the chemoattractive capacity of endothelial cells, facilitating the recruitment of circulating monocytes into the bone marrow (79, 80). Endothelial activation mediated by these miRs leads to overproduction of cytokines and growth factors such as IL-6, IL-8, MCP-1 (CCL2), GM-CSF, and G-CSF (81). These factors not only attract monocytes from the circulation but also promote their proliferation and differentiation into osteoclast precursors. This local environment further induces the expression of M-CSF and RANKL, thereby sustaining osteoclast differentiation and survival.

Taken together, these processes foster the establishment of a pro-osteoclastogenic and pro-inflammatory microenvironment, capable of amplifying osteoclast formation and activity even in the absence of exogenous stimuli, thereby supporting the concept of SO in the bone marrow mediated by P-EXOs.

Although these five signaling routes (i, ii, iii, iv, v) are described separately for conceptual clarity (Figure 1), several evidences suggest that they operate in a tightly interconnected manner within the bone marrow microenvironment. Rather than functioning as isolated cascades, P-EXO-miRs form a dynamic communication network in which each pathway can modulate or amplify the others through cytokine signaling, transcriptional feedback, and reciprocal regulation of target cells. For instance, P-EXO-miR-155–induced activation of macrophages promotes the secretion of TNF-α and IL-1β, which in turn stimulate stromal and endothelial cells to increase RANKL expression, thereby enhancing osteoclast precursor differentiation. These cytokines may also upregulate adhesion molecules such as VCAM-1 and ICAM-1, indirectly favoring further P-EXO homing and uptake, thus establishing a reinforcing loop between inflammation and exosomal signaling. Likewise, miR-21 and miR-223 may synergistically activate NFATc1 and c-Fos, driving a sustained osteoclastogenic phenotype, while miR-214 suppresses osteoblast-derived OPG, further skewing the RANKL/OPG balance toward resorption. In parallel, exosomal miR-146a and miR-27a, known regulators of NF-κB and Wnt pathways, could act as modulators that fine-tune these signals, preventing excessive bone destruction yet maintaining a chronic low-grade activation state. The overall outcome is a self-amplifying molecular network in which P-EXO-miRs not only initiate but also perpetuate a pro-osteoclastogenic milieu.

This integrative framework suggests that P-EXOs could act as central orchestrators linking inflammation, cellular crosstalk, and gene-regulatory feedback, ultimately shaping the microenvironment that favors SO.

## 4 Discussion and future perspectives

The hypothesis that P-EXO-miRs act as modulators of SO in osteoporosis offers a novel perspective on the platelet–bone axis. Previous studies have largely emphasized the contribution of pro-inflammatory cytokines and immune dysregulation in osteoclast-driven bone loss, while the role of platelet exosomes has remained unexplored (82). The proposed framework integrates hematologic, immune, stromal, and endothelial pathways, thereby extending current views of osteoporosis pathophysiology toward a more complex and intercellularly orchestrated model. To strengthen the robustness of this hypothesis, a search of the literature was conducted using PubMed, Scopus, and Web of Science (2000–2025) with the terms ‘platelet exosomes,’ ‘osteoclastogenesis,’ ‘miRNA,’ and ‘osteoporosis.’ Only peer-reviewed articles focusing on human or mammalian models were considered.

**FIGURE 2:**
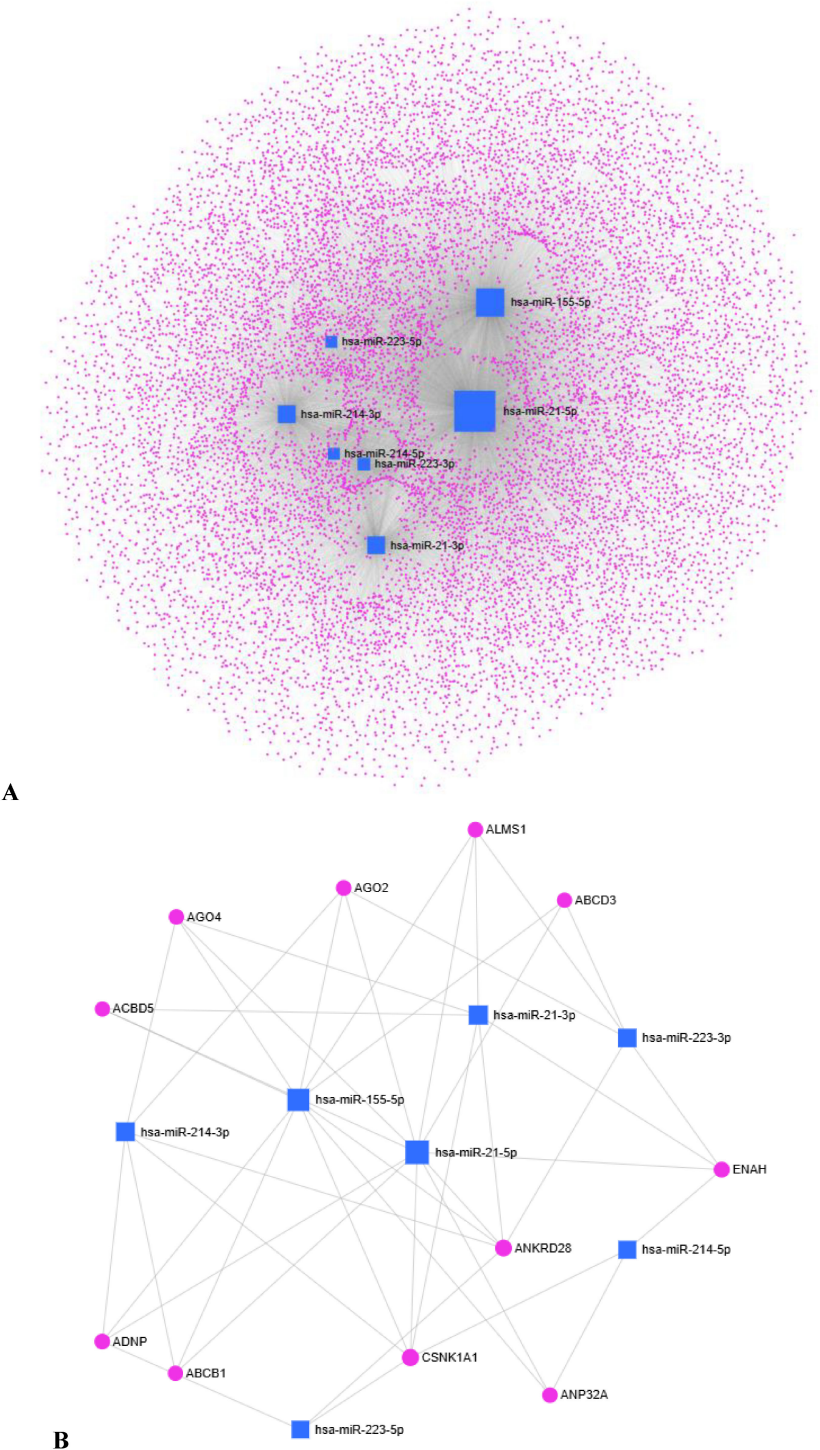
Interaction network of key P-EXO-miRs analyzed using miRNet. Blue squares represent miRs, and pink nodes indicate predicted target genes. **(A)** Complete network of interactions. **(B)** Reduced network highlighting the principal hub genes, ACBD5, AGO2, AGO4, ABCD3, ABCB1, ALMS1, ADNP, ANKRD28, ENAH, CSNK1A1, ANP32A, which are involved in key signaling pathways, including the Hedgehog and Wnt pathways and the regulation of the actin cytoskeleton.

From a mechanistic standpoint, P-EXO-miRs may exert pleiotropic effects: (i) direct priming of osteoclast precursors through the suppression of inhibitory checkpoints and induction of canonical osteoclastogenic transcription factors; (ii) alteration of the RANKL/OPG axis in stromal and osteoblastic cells via miR-214, simultaneously impairing bone formation and removing restraints on osteoclast activation; (iii) induction of low-grade chronic inflammation through miR-155–mediated macrophage polarization and cytokine release; (iv) immune system reprogramming, whereby neutrophils, T cells, and B cells acquire pro-osteoclastogenic phenotypes; and (v) endothelial activation, establishing a chemoattractive and growth factor–rich niche that sustains osteoclast precursor recruitment and differentiation. The convergence of these mechanisms provides a biologically plausible explanation for the phenomenon of SO in osteoporosis, even in the absence of classical exogenous stimuli. However, to further clarify the regulatory landscape involving the principal P-EXO-miRs identified in our hypothesis (miR-21, miR-155, miR-214, miR-223), an integrated platform linking miRNA tools, miRNet (version 2.0)^1^, was used to predict miR–gene interactions (83). The miR–gene interaction data were collected from miRTarBase v9.0 using miRBase IDs. In the network, miRs (blue squares) are connected to their predicted target genes (pink circles), revealing a dense and complex web of interactions (Figure 2A). By reducing network complexity, 11 genes, such as ACBD5, AGO2, AGO4, ABCD3, ABCB1, ALMS1, ADNP, ANKRD28, ENAH, CSNK1A1, ANP32A, emerged as central nodes (Figure 2B). These hub genes are involved in critical signaling pathways, including the Hedgehog and Wnt pathways and regulation of the actin cytoskeleton, all of which are known to contribute to osteoclast differentiation and activity. While some of the hub genes may influence these processes indirectly, the network underscores their central role in regulating miR-mediated signaling relevant to osteoclast function.

Despite its conceptual appeal, this hypothesis requires rigorous validation. One challenge lies in the specificity of platelet-derived exosomes: while platelets represent a major source of circulating vesicles, other blood and marrow cells also release miR-containing EVs, which may confound attribution of effects to P-EXOs alone. Furthermore, the heterogeneity of exosomal cargo depending on the nature of platelet activation (e.g., aging, metabolic dysregulation, inflammatory triggers) adds complexity to experimental design. Another limitation is the current paucity of *in vivo* data directly linking P-EXO-miRs to bone loss or fracture risk. In addition to these limitations, several key questions remain unanswered: Why should these specific P-EXO-miRs (e.g., miR-21, miR-155, miR-214, miR-223) preferentially activate osteoclastogenic pathways, while other platelet-derived miRs appear less involved? Do platelets possess molecular machinery to selectively process and mature these miRs, and if so, what determines this specificity? Which specific alterations in the osteoporotic bone marrow microenvironment act as triggers for platelet activation and the release of osteoclastogenesis-promoting exosomes? Could non-platelet-derived vesicles carrying overlapping miRs confound the attribution of osteoclastogenic effects to P-EXOs alone?

Future studies should address these gaps by employing a multilayered approach. At the molecular level, next-generation sequencing of P-EXO-miRs in osteoporotic versus healthy individuals could identify disease-specific signatures. Functionally, *in vitro* co-culture systems incorporating osteoclast precursors, osteoblasts, immune cells, and endothelial cells could help dissect the cell-specific uptake and effects of P-EXO-miRs. *In vivo*, mouse models of platelet depletion or exosome inhibition, combined with genetic manipulation of candidate miRs (e.g., miR-21, miR-155, miR-214, miR-223), may clarify causality. Finally, translational studies are warranted to explore whether P-EXO-miR profiles correlate with bone mineral density, fracture incidence, or response to anti-resorptive therapy. Importantly, it may also be fundamental to evaluate the effect and behavior of P-EXO-miRs in relation to specific lifestyle factors. For instance, cigarette smoking has been shown to alter circulating miR profiles, contributing to impaired bone metabolism and increased fracture risk (84). Similarly, visceral obesity is associated with altered platelet-derived miR expression (e.g., increased miR-19a), leading to enhanced platelet activation and pro-inflammatory signaling (85). A high-fat diet has also been linked to dysregulated platelet activity and changes in circulating miRs involved in lipid metabolism and inflammation (86). Conversely, regular aerobic exercise may beneficially modulate platelet miR profiles (such as miR-223), improving platelet reactivity and reducing thrombotic potential in individuals with metabolic disorders (87). From a therapeutic standpoint, the platelet-bone exosome axis may represent both a diagnostic biomarker source and a novel intervention target. Circulating P-EXO-miRs could be exploited as minimally invasive indicators of osteoclastogenic potential or disease activity. Conversely, strategies to modulate P-EXO-miR release, block their uptake, or selectively inhibit key miRs might restore the balance between bone formation and resorption. Such approaches could complement existing anti-resorptive and anabolic therapies, particularly in patients with inflammatory or platelet-related comorbidities.

In conclusion, the proposed role of P-EXO-miRs in SO highlights an unrecognized dimension of osteoimmunology. By bridging platelet biology, inflammation, and skeletal remodeling, this framework not only enriches our understanding of osteoporosis but also paves the way for innovative diagnostic and therapeutic opportunities.

## Data Availability

The original contributions presented in this study are included in this article/supplementary material, further inquiries can be directed to the corresponding author.

## Footnotes

1
http://www.mirnet.ca

## References

[1] van Oostwaard M. Osteoporosis and the nature of fragility fracture: An overview. In: Hertz K Santy-Tomlinson J editors. *Fragility Fracture Nursing: Holistic Care and Management of the Orthogeriatric Patient.* Cham, CH: Springer (2018). 10.1007/978-3-319-76681-2_1

[2] NIH. Available online at: https://www.niams.nih.gov/health-topics/osteoporosis (accessed March 3, 2025). (2025)

[3] Keen M Barnett M Anastasopoulou C. *Osteoporosis in Females.* Treasure Island, FL: StatPearls Publishing (2025). 32644582

[4] Willers C Norton N Harvey N Jacobson T Johansson H Lorentzon M Osteoporosis in Europe: a compendium of country-specific reports. *Arch Osteoporos.* (2022) 17:23. 10.1007/s11657-021-00969-8 35079919 PMC8789736

[5] Tao R Liu C Wong P Huang T Alt V Rupp M Advances in immune mechanisms and developing immune-targeted therapies for osteoporosis: a systematic review. *Pharmacol Res.* (2025) 218:107835. 10.1016/j.phrs.2025.107835 40550406

[6] Saxena Y Routh S Mukhopadhaya A. Immunoporosis: role of innate immune cells in osteoporosis. *Front Immunol.* (2021) 12:687037. 10.3389/fimmu.2021.687037 34421899 PMC8374941

[7] Iantomasi T Romagnoli C Palmini G Donati S Falsetti I Miglietta F Oxidative stress and inflammation in osteoporosis: molecular mechanisms involved and the relationship with microRNAs. *Int J Mol Sci.* (2023) 24:3772. 10.3390/ijms24043772 36835184 PMC9963528

[8] Salamanna F Maglio M Giavaresi G Pagani S Giardino R Fini M. In vitro method for the screening and monitoring of estrogen-deficiency osteoporosis by targeting peripheral circulating monocytes. *Age.* (2015) 37:9819. 10.1007/s11357-015-9819-4 26250906 PMC5005821

[9] Salamanna F Maglio M Borsari V Giavaresi G Aldini N Fini M. Peripheral Blood mononuclear cells spontaneous osteoclastogenesis: mechanisms driving the process and clinical relevance in skeletal disease. *J Cell Physiol.* (2016) 231:521–30. 10.1002/jcp.25134 26284737

[10] Salamanna F Giardino R Fini M. Spontaneous osteoclastogenesis: hypothesis for gender-unrelated osteoporosis screening and diagnosis. *Med Hypotheses.* (2017) 109:70–2. 10.1016/j.mehy.2017.09.028 29150298

[11] Salamanna F Di Martino A Contartese D Faldini C Giavaresi G Fini M. Correction: the unexplored relationship between spontaneous osteoclastogenesis and platelets in osteoporosis. *Front Med.* (2025) 12:1670375. 10.3389/fmed.2025.1670375 40837561 PMC12361230

[12] Salamanna F Maglio M Sartori M Tschon M Fini M. Platelet features and derivatives in osteoporosis: a rational and systematic review on the best evidence. *Int J Mol Sci.* (2020) 21:1762. 10.3390/ijms21051762 32143494 PMC7084230

[13] Oliveri C Xourafa A Agostino R Corigliano V Botindari A Gaudio A Exploring the association between platelet count, the systemic immune inflammation index, and fracture risk in postmenopausal women with osteoporosis: a cross-sectional study. *J Clin Med.* (2025) 14:5453. 10.3390/jcm14155453 40807074 PMC12347221

[14] Nurden A. The biology of the platelet with special reference to inflammation, wound healing and immunity. *Front Biosci.* (2018) 23:726–51. 10.2741/4613 28930569

[15] Repsold L Joubert A. Platelet function, role in thrombosis, inflammation, and consequences in chronic myeloproliferative disorders. *Cells.* (2021) 10:3034. 10.3390/cells10113034 34831257 PMC8616365

[16] Yun S Sim E Goh R Park J Han J. Platelet activation: the mechanisms and potential biomarkers. *Biomed Res Int.* (2016) 2016:9060143. 10.1155/2016/9060143 27403440 PMC4925965

[17] Tao S Guo S Zhang C. Platelet-derived extracellular vesicles: an emerging therapeutic approach. *Int J Biol Sci.* (2017) 13:828–34. 10.7150/ijbs.19776 28808416 PMC5555101

[18] Landry P Plante I Ouellet D Perron M Rousseau G Provost P. Existence of a microRNA pathway in anucleate platelets. *Nat Struct Mol Biol.* (2009) 16:961–6. 10.1038/nsmb.1651 19668211 PMC2911476

[19] Ed Nignpense B Chinkwo K Blanchard C Santhakumar A. Polyphenols: modulators of platelet function and platelet microparticle generation? *Int J Mol Sci.* (2019) 21:146. 10.3390/ijms21010146 31878290 PMC6981839

[20] Plé H Landry P Benham A Coarfa C Gunaratne P Provost P. The repertoire and features of human platelet microRNAs. *PLoS One.* (2012) 7:e50746. 10.1371/journal.pone.0050746 23226537 PMC3514217

[21] Bray P McKenzie S Edelstein L Nagalla S Delgrosso K Ertel A The complex transcriptional landscape of the anucleate human platelet. *BMC Genomics.* (2013) 14:1. 10.1186/1471-2164-14-1 23323973 PMC3722126

[22] Blair P Flaumenhaft R. Platelet alpha-granules: basic biology and clinical correlates. *Blood Rev.* (2009) 23:177–89. 10.1016/j.blre.2009.04.001 19450911 PMC2720568

[23] Heijnen H Schiel A Fijnheer R Geuze H Sixma J. Activated platelets release two types of membrane vesicles: microvesicles by surface shedding and exosomes derived from exocytosis of multivesicular bodies and alpha-granules. *Blood.* (1999) 94:3791–9. 10572093

[24] Dupuis A Bordet J Eckly A Gachet C. Platelet δ-storage pool disease: an update. *J Clin Med.* (2020) 9:2508. 10.3390/jcm9082508 32759727 PMC7466064

[25] King S Reed G. Development of platelet secretory granules. *Semin Cell Dev Biol.* (2002) 13:293–302. 10.1016/s1084952102000599 12243729

[26] Krammer T Mayr M Hackl M. microRNAs as promising biomarkers of platelet activity in antiplatelet therapy monitoring. *Int J Mol Sci.* (2020) 21:3477. 10.3390/ijms21103477 32423125 PMC7278969

[27] Arraud N Linares R Tan S Gounou C Pasquet J Mornet S Extracellular vesicles from blood plasma: determination of their morphology, size, phenotype and concentration. *J Thromb Haemost.* (2014) 12:614–27. 10.1111/jth.12554 24618123

[28] Zaldivia M McFadyen J Lim B Wang X Peter K. Platelet-derived microvesicles in cardiovascular diseases. *Front Cardiovasc Med.* (2017) 4:74. 10.3389/fcvm.2017.00074 29209618 PMC5702324

[29] Yeh H Gupta K Lu Y Srinivasan A Delila L Yen N Platelet extracellular vesicles as natural delivery vehicles for mitochondrial dysfunction therapy? *ACS Biomater Sci Eng.* (2025) 11:2601–21. 10.1021/acsbiomaterials.5c00473 40280866 PMC12076291

[30] van Niel G D’Angelo G Raposo G. Shedding light on the cell biology of extracellular vesicles. *Nat Rev Mol Cell Biol.* (2018) 19:213–28. 10.1038/nrm.2017.125 29339798

[31] Hurley J. ESCRTs are everywhere. *EMBO J.* (2015) 34:2398–407. 10.15252/embj.201592484 26311197 PMC4601661

[32] Jankovičová J Sečová P Michalková K Antalíková J. Tetraspanins, more than markers of extracellular vesicles in reproduction. *Int J Mol Sci.* (2020) 21:7568. 10.3390/ijms21207568 33066349 PMC7589920

[33] Quévillon Huberdeau M Simard MJ. A guide to microRNA-mediated gene silencing. *FEBS J.* (2019) 286:642–52. 10.1111/febs.14666 30267606

[34] Laffont B Corduan A Plé H Duchez A Cloutier N Boilard E Activated platelets can deliver mRNA regulatory Ago2 microRNA complexes to endothelial cells via microparticles. *Blood.* (2013) 122:253–61. 10.1182/blood-2013-03-492801 23652806

[35] Li M Li S Du C Zhang Y Li Y Chu L Exosomes from different cells: characteristics, modifications, and therapeutic applications. *Eur J Med Chem.* (2020) 207:112784. 10.1016/j.ejmech.2020.112784 33007722

[36] Edelstein L. The role of platelet microvesicles in intercellular communication. *Platelets.* (2017) 28:222–7. 10.1080/09537104.2016.1257114 27928930

[37] Muttiah B Ng S Lokanathan Y Ng M Law J. Beyond blood clotting: the many roles of platelet-derived extracellular vesicles. *Biomedicines.* (2024) 12:1850. 10.3390/biomedicines12081850 39200314 PMC11351396

[38] Geng Z Sun T Yuan L Zhao Y. The existing evidence for the use of extracellular vesicles in the treatment of osteoporosis: a review. *Int J Surg.* (2025) 111:3414–29. 10.1097/JS9.0000000000002339 40085758 PMC12165590

[39] Tang P Xiong Q Ge W Zhang L. The role of microRNAs in osteoclasts and osteoporosis. *RNA Biol.* (2014) 11:1355–63. 10.1080/15476286.2014.996462 25692234 PMC4615571

[40] Sanwlani R Gangoda L. Role of extracellular vesicles in cell death and inflammation. *Cells.* (2021) 10:2663. 10.3390/cells10102663 34685643 PMC8534608

[41] Livshits G Kalinkovich A. Targeting chronic inflammation as a potential adjuvant therapy for osteoporosis. *Life Sci.* (2022) 306:120847. 10.1016/j.lfs.2022.120847 35908619

[42] Singh V Kaur R Kumari P Pasricha C Singh R. ICAM-1 and VCAM-1: gatekeepers in various inflammatory and cardiovascular disorders. *Clin Chim Acta.* (2023) 548:117487. 10.1016/j.cca.2023.117487 37442359

[43] Fatehi F Mollahosseini M Hassanshahi G Khanamani Falahati-Pour S Khorramdelazad H Ahmadi Z CC chemokines CCL2, CCL3, CCL4 and CCL5 are elevated in osteoporosis patients. *J Biomed Res.* (2017) 31:468–70. 10.7555/JBR.31.20150166 28958999 PMC5706441

[44] Saumell-Esnaola M Delgado D García Del Caño G Beitia M Sallés J González-Burguera I. Isolation of platelet-derived exosomes from human platelet-rich plasma: biochemical and morphological characterization. *Int J Mol Sci.* (2022) 23:2861. 10.3390/ijms23052861 35270001 PMC8911307

[45] Weiss R Gröger M Rauscher S Fendl B Eichhorn T Fischer M Differential interaction of platelet-derived extracellular vesicles with leukocyte subsets in human whole blood. *Sci Rep.* (2018) 8:6598. 10.1038/s41598-018-25047-x 29700367 PMC5920058

[46] Sugatani T Vacher J Hruska KA. A microRNA expression signature of osteoclastogenesis. *Blood.* (2011) 117:3648–57. 10.1182/blood-2010-10-311415 21273303 PMC3072882

[47] Talotta F Cimmino A Matarazzo M Casalino L De Vita G D’Esposito M An autoregulatory loop mediated by miR-21 and PDCD4 controls the AP-1 activity in RAS transformation. *Oncogene.* (2009) 28:73–84. 10.1038/onc.2008.370 18850008

[48] Thum T Gross C Fiedler J Fischer T Kissler S Bussen M MicroRNA-21 contributes to myocardial disease by stimulating MAP kinase signalling in fibroblasts. *Nature.* (2008) 456:980–4. 10.1038/nature07511 19043405

[49] Inoue K Ng C Xia Y Zhao B. Regulation of osteoclastogenesis and bone resorption by miRNAs. *Front Cell Dev Biol.* (2021) 9:651161. 10.3389/fcell.2021.651161 34222229 PMC8249944

[50] Xu J Yu L Liu F Wan L Deng Z. The effect of cytokines on osteoblasts and osteoclasts in bone remodeling in osteoporosis: a review. *Front Immunol.* (2023) 14:1222129. 10.3389/fimmu.2023.1222129 37475866 PMC10355373

[51] Dang C Leelahavanichkul A. Over-expression of miR-223 induces M2 macrophage through glycolysis alteration and attenuates LPS-induced sepsis mouse model, the cell-based therapy in sepsis. *PLoS One.* (2020) 15:e0236038. 10.1371/journal.pone.0236038 32658933 PMC7357756

[52] Nguyen M Hoang H Rasheed A Duchez A Wyatt H Cottee M miR-223 exerts translational control of proatherogenic genes in macrophages. *Circ Res.* (2022) 131:42–58. 10.1161/CIRCRESAHA.121.319120 35611698 PMC9213086

[53] Lozano C Duroux-Richard I Firat H Schordan E Apparailly F. MicroRNAs: key regulators to understand osteoclast differentiation? *Front Immunol.* (2019) 10:375. 10.3389/fimmu.2019.00375 30899258 PMC6416164

[54] Behera J Tyagi N. Exosomes: mediators of bone diseases, protection, and therapeutics potential. *Oncoscience.* (2018) 5:181–95. 10.18632/oncoscience.421 30035185 PMC6049320

[55] Li K Chang Y Hsu M Lo S Li W Hu Y. Baculovirus-mediated miR-214 knockdown shifts osteoporotic ASCs differentiation and improves osteoporotic bone defects repair. *Sci Rep.* (2017) 7:16225. 10.1038/s41598-017-16547-3 29176755 PMC5701180

[56] Xie X Xiong Y Panayi A Hu L Zhou W Xue H Exosomes as a novel approach to reverse osteoporosis: a review of the literature. *Front Bioeng Biotechnol.* (2020) 8:594247. 10.3389/fbioe.2020.594247 33195163 PMC7644826

[57] Pasca S Jurj A Petrushev B Tomuleasa C Matei D. MicroRNA-155 implication in M1 polarization and the impact in inflammatory diseases. *Front Immunol.* (2020) 11:625. 10.3389/fimmu.2020.00625 32351507 PMC7174664

[58] Chen S Saeed A Liu Q Jiang Q Xu H Xiao G Macrophages in immunoregulation and therapeutics. *Signal Transduct Target Ther.* (2023) 8:207. 10.1038/s41392-023-01452-1 37211559 PMC10200802

[59] Hu Y Huang J Chen C Wang Y Hao Z Chen T Strategies of macrophages to maintain bone homeostasis and promote bone repair: a narrative review. *J Funct Biomater.* (2022) 14:18. 10.3390/jfb14010018 36662065 PMC9864083

[60] Zheng Z Wu L Li Z Tang R Li H Huang Y Mir155 regulates osteogenesis and bone mass phenotype via targeting S1pr1 gene. *Elife.* (2023) 12:e77742. 10.7554/eLife.77742 36598122 PMC9839347

[61] Wu T Xie M Wang X Jiang X Li J Huang H. miR-155 modulates TNF-α-inhibited osteogenic differentiation by targeting SOCS1 expression. *Bone.* (2012) 51:498–505. 10.1016/j.bone.2012.05.013 22634176

[62] Ye J Guo R Shi Y Qi F Guo C Yang L. miR-155 regulated inflammation response by the SOCS1-STAT3-PDCD4 axis in atherogenesis. *Mediators Inflamm.* (2016) 2016:8060182. 10.1155/2016/8060182 27843203 PMC5098093

[63] Sheedy F Palsson-McDermott E Hennessy E Martin C O’Leary J Ruan Q Negative regulation of TLR4 via targeting of the proinflammatory tumor suppressor PDCD4 by the microRNA miR-21. *Nat Immunol.* (2010) 11:141–7. 10.1038/ni.1828 19946272

[64] Saadh M Saeed T Alfarttoosi K Sanghvi G Roopashree R Thakur V Exosomes and MicroRNAs: key modulators of macrophage polarization in sepsis pathophysiology. *Eur J Med Res.* (2025) 30:298. 10.1186/s40001-025-02561-z 40247413 PMC12007276

[65] Kumar M Baba S Sadida H Marzooqi S Jerobin J Altemani F Extracellular vesicles as tools and targets in therapy for diseases. *Signal Transduct Target Ther.* (2024) 9:27. 10.1038/s41392-024-01735-1 38311623 PMC10838959

[66] Luo J Li L Shi W Xu K Shen Y Dai B. Oxidative stress and inflammation: roles in osteoporosis. *Front Immunol.* (2025) 16:1611932. 10.3389/fimmu.2025.1611932 40873591 PMC12379731

[67] Domazetovic V Marcucci G Iantomasi T Brandi M Vincenzini M. Oxidative stress in bone remodeling: role of antioxidants. *Clin Cases Miner Bone Metab.* (2017) 14:209–16. 10.11138/ccmbm/2017.14.1.209 29263736 PMC5726212

[68] Sato K Suematsu A Okamoto K Yamaguchi A Morishita Y Kadono Y Th17 functions as an osteoclastogenic helper T cell subset that links T cell activation and bone destruction. *J Exp Med.* (2006) 203:2673–82. 10.1084/jem.20061775 17088434 PMC2118166

[69] Cheng X Chen Y Zhou X Gu Q Zhao H Wan C Immunoporosis: the hidden link between aging immune cells and bone fragility. *J Orthop Translat.* (2025) 53:325–35. 10.1016/j.jot.2025.06.015 40933229 PMC12417214

[70] Ivanov I Zhou L Littman D. Transcriptional regulation of Th17 cell differentiation. *Semin Immunol.* (2007) 19:409–17. 10.1016/j.smim.2007.10.011 18053739 PMC2696342

[71] Wang W Qiao S Kong X Zhang G Cai Z. The role of exosomes in immunopathology and potential therapeutic implications. *Cell Mol Immunol.* (2025) 22:975–95. 10.1038/s41423-025-01323-5 40659888 PMC12398553

[72] Nikolajczyk BS. B cells as under-appreciated mediators of non-auto-immune inflammatory disease. *Cytokine.* (2010) 50:234–42. 10.1016/j.cyto.2010.02.022 20382544 PMC2917985

[73] Jiao P Wang X Luoreng Z Yang J Jia L Ma Y miR-223: an Effective Regulator of Immune Cell Differentiation and Inflammation. *Int J Biol Sci.* (2021) 17:2308–22. 10.7150/ijbs.59876 34239357 PMC8241730

[74] Sugatani T Hruska K. MicroRNA-223 is a key factor in osteoclast differentiation. *J Cell Biochem.* (2007) 101:996–9. 10.1002/jcb.21335 17471500

[75] Oka S Li X Zhang F Tewari N Ma R Zhong L MicroRNA-21 facilitates osteoblast activity. *Biochem Biophys Rep.* (2020) 25:100894. 10.1016/j.bbrep.2020.100894 33426313 PMC7782325

[76] Sheedy F Grebe A Rayner K Kalantari P Ramkhelawon B Carpenter S CD36 coordinates NLRP3 inflammasome activation by facilitating intracellular nucleation of soluble ligands into particulate ligands in sterile inflammation. *Nat Immunol.* (2013) 14:812–20. 10.1038/ni.2639 23812099 PMC3720827

[77] O’Connell R Rao D Baltimore D. microRNA regulation of inflammatory responses. *Annu Rev Immunol.* (2012) 30:295–312. 10.1146/annurev-immunol-020711-075013 22224773

[78] Li Y Tian L Yang Z Liu Y Li D Tang Z. miR-155 targets SOCS1 to modulate the phenotype transition of M1 macrophage in distraction osteogenesis promoted by PTH administration. *Eur J Med Res.* (2025) 30:438. 10.1186/s40001-025-02683-4 40450308 PMC12125726

[79] LiuS WangM XuL DengD LuL TianJ New insight into the role of SOCS family in immune regulation and autoimmune pathogenesis. *J Adv Res.* (2025): 10.1016/j.jare.2025.05.020 Online ahead of print.40349956

[80] Guo B Gu J Zhuang T Zhang J Fan C Li Y MicroRNA-126: from biology to therapeutics. *Biomed Pharmacother.* (2025) 185:117953. 10.1016/j.biopha.2025.117953 40036996

[81] Chakraborty C Sharma A Sharma G Lee S. The interplay among miRNAs, major cytokines, and cancer-related inflammation. *Mol Ther Nucleic Acids.* (2020) 20:606–20. 10.1016/j.omtn.2020.04.002 32348938 PMC7191126

[82] Wang Y Zheng S Luo Y Xiao W Huang C Li Y. Osteoimmunology and aging: mechanisms, implications, and therapeutic perspectives. *Ageing Res Rev.* (2025) 111:102822. 10.1016/j.arr.2025.102822 40609646

[83] Chang L Zhou G Soufan O Xia J. miRNet 2.0: network-based visual analytics for miRNA functional analysis and systems biology. *Nucleic Acids Res.* (2020) 48:W244–51. 10.1093/nar/gkaa467 32484539 PMC7319552

[84] Willinger C Rong J Tanriverdi K Courchesne P Huan T Wasserman G MicroRNA signature of cigarette smoking and evidence for a putative causal role of MicroRNAs in smoking-related inflammation and target organ damage. *Circ Cardiovasc Genet.* (2017) 10:e001678. 10.1161/CIRCGENETICS.116.001678 29030400 PMC5683429

[85] Cariello M Piccinin E Pasculli E Arconzo M Zerlotin R D’Amore S Platelets from patients with visceral obesity promote colon cancer growth. *Commun Biol.* (2022) 5:553. 10.1038/s42003-022-03486-7 35672444 PMC9174292

[86] Das D Shruthi N Banerjee A Jothimani G Duttaroy A Pathak S. Endothelial dysfunction, platelet hyperactivity, hypertension, and the metabolic syndrome: molecular insights and combating strategies. *Front Nutr.* (2023) 10:1221438. 10.3389/fnut.2023.1221438 37614749 PMC10442661

[87] Taghizadeh M Kargarfard M Braune S Jung F Naderi M. Long-term aerobic exercise training in type two diabetic patients alters the expression of miRNA-223 and its corresponding target, the P2RY12 receptor, attenuating platelet function. *Clin Hemorheol Microcirc.* (2022) 80:107–16. 10.3233/CH-211209 34420942

