# Correction: How exosomal platelet-derived miRNAs can lead to spontaneous osteoclastogenesis in osteoporosis: a new mechanistic viewpoint

**DOI:** 10.3389/fmed.2025.1763866

**Published:** 2026-01-09

**Authors:** Francesca Salamanna, Gianluca Giavaresi, Alberto Di Martino, Agostino Gaudio, Fabiana Nucera, Cesare Faldini, Milena Fini

**Affiliations:** 1 Surgical Sciences and Technologies, IRCCS Istituto Ortopedico Rizzoli, Bologna, Italy; 2 1st Orthopaedic and Traumatologic Clinic, IRCCS Istituto Ortopedico Rizzoli, Bologna, Italy; 3 Department of Biomedical and Neuromotor Science-DIBINEM, University of Bologna, Bologna, Italy; 4 Department of Clinical and Experimental Medicine, University of Catania, Catania, Italy; 5 Scientific Direction, IRCCS Istituto Ortopedico Rizzoli, Bologna, Italy

**Keywords:** exosomal platelet-derived miRNAs, spontaneous osteoclastogenesis, osteoporosis, platelet, platelet-bone-axis

An incorrect **Funding** statement was provided. It originally stated: The author(s) declare financial support was received for the research and/or publication of this article. This study was funded by the Italian Ministry of Health in the PNRR: M6/C2_CALL 2023 (PNRR-POC-2023-12378461) “Sustainable Innovation with a Method Based on Peripheral Mononuclear Cells to Screen, Monitor and Stratify the Population at Risk of Osteoporosis and Fractures – DISCERN.”

The correct **Funding** statement is:

The author(s) declared that financial support was received for this work and/or its publication. This study was funded by Finanziamento erogato nel rispetto della Missione 6/Componente 2/Investimento 2.1 “Rafforzamento e potenziamento della ricerca biomedica del SSN”, finanziato dall'Unione europea – NextGenerationEU-PNRR-POC-2023-12378461—Sustainable Innovation with a Method Based on Peripheral Mononuclear Cells to Screen, Monitor, and Stratify the Population at Risk of Osteoporosis and Fractures—Discern CUP code: D33C24000440006.

The original version of this article has been updated.

